# The Value of Optical Coherence Tomography in Patients with Pituitary Adenoma and Its Association with Clinical Features: A Pilot Study

**DOI:** 10.3390/jcm14124318

**Published:** 2025-06-17

**Authors:** Monika Duseikaite, Alvita Vilkeviciute, Igne Dumbliauskaite, Brigita Glebauskiene, Indre Zostautiene, Vita Rovite, Sheng-Nan Wu, Arimantas Tamasauskas, Rasa Liutkeviciene

**Affiliations:** 1 Laboratory of Ophthalmology, Institute of Neuroscience, Lithuanian University of Health Sciences, Eivenių Str. 2, LT-50161 Kaunas, Lithuania; monika.duseikaite@lsmu.lt (M.D.); rasa.liutkeviciene@lsmu.lt (R.L.); 2 Medical Academy, Lithuanian University of Health Sciences, Eivenių Str. 2, LT-50161 Kaunas, Lithuania; igne.dumbliauskaite@stud.lsmu.lt; 3 Department of Ophthalmology, Medical Academy, Lithuanian University of Health Sciences, Eivenių Str. 2, LT-50161 Kaunas, Lithuania; brigita.glebauskiene@lsmuni.lt; 4 Radiology Department, Medicine Academy, Lithuanian University of Health Sciences, Eivenių Str. 2, LT-50161 Kaunas, Lithuania; indre.zostautiene@kaunoklinikos.lt; 5 Latvian Biomedical Research and Study Centre, Ratsupites Str. 1-k1, LV-1067 Riga, Latvia; vita.rovite@biomed.lu.lv; 6 Department of Neurology, National Cheng Kung University Hospital, Tainan City 704, Taiwan; snwu@ncku.edu.tw; 7 Department of Neurosurgery, Hospital of Lithuanian University of Health Sciences, Kaunas Clinics, LT-50161 Kaunas, Lithuania; arimantas.tamasauskas@lsmu.lt

**Keywords:** pituitary adenoma, optical coherence tomography, MRI, RNFL, GCL+, GCL++

## Abstract

**Background**: The main mechanism of optic nerve damage in patients with pituitary adenoma (PA) is the pressure of optic chiasm. The retinal nerve fiber layer (RNFL), the ganglion cell layer (GCL)+, and GCL++ thickness measurement by optical coherence tomography (OCT), visual function evaluation, and magnetic resonance imaging (MRI) can be used to predict visual function recovery. In our study, we investigated the associations between visual acuity (VA), visual field (VF), RNFL, GCL changes, and the findings of MRI in patients with PA. **Methods**: This study was conducted in the Departments of Ophthalmology and Neurosurgery of the Lithuanian University of Health Sciences Hospital. A total of 25 patients diagnosed with PA were included in the study group, and 27 healthy subjects were included in the control group. The thickness of the RNFL and ganglion cell layer (GCL+, GCL++) and optic nerve disc diameter was analysed with OCT. Moreover, an MRI was performed for patients with PA. **Results**: The RNFL thickness around the optic disk measured preoperatively was reduced significantly in the temporal quadrant in PA patients compared with the control group (median (min; max); mean rank: 73.5 (52; 109); 58.39 vs. 69.5 (16; 168); 46.14; *p* = 0.038). We found that it was reduced significantly only in the inferior quadrant of the macro-PA group compared to the micro-PA group (median (min; max); mean rank: 99.5 (61; 115); 21.07 vs. 106.5 (90; 121); 32.15), *p* = 0.008, respectively). The RNFL thickness was reduced significantly only in the inferior quadrant of the non-active PA group compared to the active PA group (median (min; max); mean rank: 118.5 (49; 144); 17.42 vs. 130.5 (77; 156); 28.05), *p* = 0.028, respectively). RNFL thickness was reduced significantly only in the temporal quadrant in the PA with suprasellar extension group compared to the PA without suprasellar extension group (median (min; max); mean rank: 67.5 (16; 99); 21.66 vs. 72 (58; 168); 30.39), *p* = 0.036, respectively). Furthermore, GCL++ thickness was reduced significantly in total and in superior and inferior sectors of the PA with suprasellar extension group compared to the PA without suprasellar extension group (median (min; max); mean rank: 98.5 (57; 113); 21.8; 101 (61; 121); 21.48 and 102.5 (59; 116); 21.71 vs. 103.5 (95; 115); 30.2; 106.5 (90; 115); 30.61 and 104.5 (95; 113); 30.32), *p* = 0.043; *p* = 0.028 and *p* = 0.038, respectively). In the control group, significant positive correlations were found between optic disc area and the total RNFL thickness (r = 0.440, *p* < 0.001). In the PA group, significant correlations were observed between optic rim area and total RNFL thickness (r = 0.493, *p* < 0.001) and all quadrants, with the strongest in the nasal quadrant (r = 0.503, *p* < 0.001). A moderate to strong negative correlation was found between visual field (VF) defects and RNFL thickness, with the strongest correlation observed in the superior quadrant. **Conclusions**: OCT offers a detailed insight into the microscopic structural and functional changes throughout the entire visual pathway in patients with PA. Our findings demonstrate a significant negative correlation between RNFL thickness and visual field defects, highlighting the clinical relevance of OCT measurements in visual function assessment. Moreover, the results suggest that optic rim area may be a more reliable indicator of RNFL thickness variations than optic disc area in patients with PA.

## 1. Introduction

Pituitary adenomas (PAs) are benign neoplasms arising from the anterior pituitary gland (adenohypophysis), with a reported prevalence rate between 14% and 23% in various autopsy and radiologic studies, representing 5% to 20% of all intracranial tumors [1,2]. These tumors are typically categorized into hormone-secreting (functioning) and nonfunctioning tumors. The hormone-secreting type includes adenomas that release various hormones, such as prolactin (lactotropes), corticotropin (corticotropes), growth hormone (somatotropes), thyroid-stimulating hormone (thyrotropes), and gonadotropins like follicle-stimulating hormone and luteinizing hormone (gonadotrophs), among others [3]. PAs are typically linked to hormone overproduction and/or symptoms of compression caused by pressure on surrounding structures, such as the optic chiasm, which can lead to progressive visual loss in one or both eyes, ultimately resulting in impaired vision, including visual field defects (46–75%) and decreased visual acuity (14–44%) [4,5]. Symptoms are related to size and growing directions of the PA; when the adenoma exceeds 10 mm in diameter (classified as a ‘macroadenoma’), it may extend beyond the boundaries of the sella turtica and can cause neuro-ophthalmological disorders by compressing the adjacent structures [6].

Various studies have found that the ganglion cell layer (GCL) can be important in evaluating optic nerve damage in patients with PA [7,8,9,10]. Retinal nerve fiber layer (RNFL) abnormalities are also a typical feature of long-standing pituitary tumors [11]. However, the use of OCT measurements alone to predict visual function is controversial. Yoneoka et al. suggested that regular RNFL thickness independently predicts postoperative visual field recovery [12]. Zhang et al. reported that PA patients with normal RNFL thickness had a significantly higher chance of visual recovery, with a combined odds ratio of 15.61 (95% CI: 4.09–59.61), compared to those patients with RNFL thinning [13]. In contrast, Póczoš et al. suggested that preoperative RNFL thinning alone is not a reliable predictor of postoperative visual improvement [10]. This controversy may be because the results obtained from RNFL changes alone do not provide a complete assessment of the entire visual pathway particularly when trying to distinguish microstructural damage.

Optic nerve disc diameter is one of the structural parameters that can also play a role in the prediction of visual functions. Therefore, the relationship between optic nerve disc (OND) size and optic nerve damage remains controversial. Several studies have found that RNFL thickness measured at a fixed diameter positively correlates with optic disc area, but the influence of OND size on RNFL thickness is not fully understood [14].

Studies have attempted to link visual acuity (VA) and visual field (VF) outcomes with OCT measurements and radiological features of chiasmal compression, but the results have been inconsistent and varied [15,16]. It has also been found that RNFL thinning does not always correspond to visual impairments or the radiological severity of chiasmal compression [17,18]. 

Our study investigated the association between VA, VF, optic nerve disc diameter, RNFL, GCL changes, and MRI results in patients with PA.

## 2. Materials and Methods

Permission to conduct the study (No. BE-2-47, 14 December 2016) was granted by the Ethics Committee for Biomedical Research. Subjects in both the PA group and the control group signed informed consent to participate in the study. The study was conducted in the Departments of Ophthalmology and Neurosurgery of the Lithuanian University of Health Sciences Hospital.

The subjects of the study were 25 cases of PA.

The PA group inclusion criteria were as follows:

1. established PA confirmed by MRI;
2. good general condition of the patient;
3. patient’s consent to participate in the study;
4. age ≥ 18 years;
5. no other brain tumors, intracranial infections, demyelinating lesions or cerebrovascular diseases;
6. no ophthalmological eye disorders detected during a detailed ophthalmological examination.

The control group inclusion criteria were as follows:

1. participants should be generally healthy, with no history of PA or major health conditions;
2. age ≥ 18 years;
3. no brain or systemic diseases;
4. consent to participate in the study.

Control group exclusion criteria included the following:

1. individuals with major health issues, including pituitary disorders, brain tumors, or serious systemic diseases;
2. those under 18, who were excluded to ensure consistency with the patient group;
3. individuals who did not give informed consent.

### 2.1. Visual Acuity and Visual Field Evaluation

Uncorrected and best-corrected VA (ranging from 0.1 to 1.0 in decimal units) were measured using Landolt rings (C optotypes) based on the Snellen test. Intraocular pressure, bio-microscopy, and fundoscopy were performed to assess corneal and lens clarity and to examine the ocular fundus. The pupils of the patients were dilated with 1% tropicamide prior to examination. Standard automated perimetry was performed using a Humphrey Field Analyser (Model 745i, Carl Zeiss Meditec Inc., Dublin, CA, USA). Although this test typically evaluates only the central 24–30° of the VF, a full-field screening (135 points, 87 degrees temporally) was conducted. During the test, each eye fixates on a central point while light stimuli of varying intensities appear in the peripheral field, and the subject responds by pressing a button when the light is seen. VF defects are measured as the number of undetected points in a 135-point full-field perimetry. A VF test was classified as unreliable if fixation loss, false negative, or false positive errors were more than 20%.

### 2.2. Invasiveness Evaluation

All PAs were analysed based on MR imaging findings. The suprasellar extension and sphenoid sinus invasion by PAs were classified according to the Wilson Hardy classification (the Hardy classification, modified by Wilson). The degree of suprasellar and parasellar extension was graded as stages A–E. The degree of sellar floor erosion was graded as grades I–IV. Grade III, localized sellar destruction, and grade IV, diffuse destruction, were considered as invasive PA. The Knosp classification system was used to quantify invasion of the cavernous sinus, in which only grades 3 and 4 define true invasion of the tumor into the cavernous sinus. Grade 0 indicates no cavernous sinus involvement; in grades 1 and 2, the tumor pushes into the medial wall of the cavernous sinus but does not go beyond a hypothetical line extending between the centers of the two segments of the internal carotid artery (grade 1), or it goes beyond such a line but without passing a line tangent to the lateral margins of the artery itself (grade 2); in grade 3, the tumor extends laterally to the internal carotid artery within the cavernous sinus; and grade 4, there is total encasement of the intracavernous carotid artery. Grade III and IV tumors were considered to be invasive.

### 2.3. Optical Coherence Tomography

**Figure 1:**
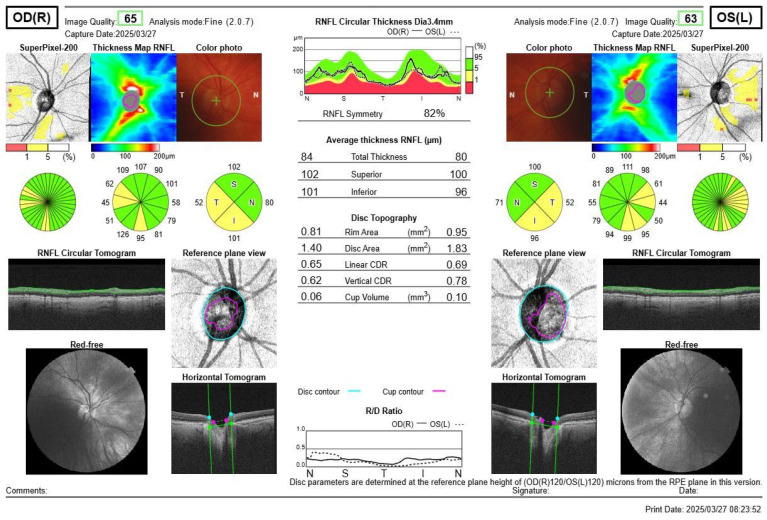
Representative OCT scan from a patient with PA. The yellow color in the thickness map highlights areas where RNFL measurements fall below normal limits, indicating potential anomalies due to optic nerve fiber damage. The circular tomograms, reference plane views, and thickness graphs provide a detailed overview of RNFL thickness across quadrants. These findings illustrate structural changes associated with chiasmal compression in PA patients.

**Figure 2:**
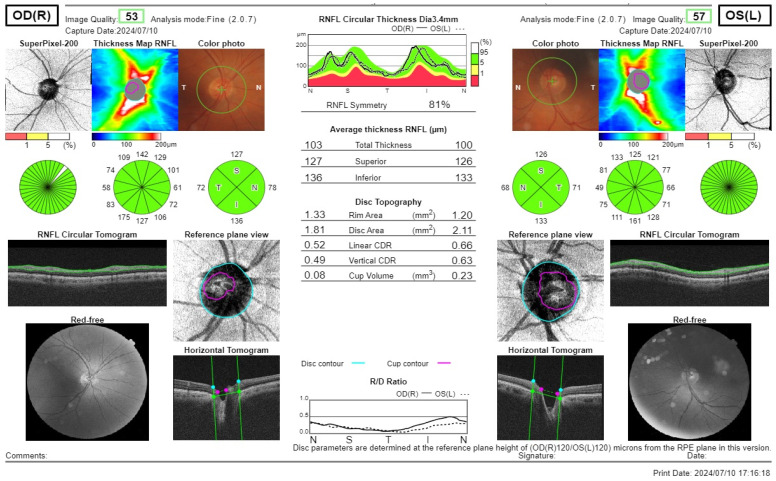
Representative OCT scans of a healthy control subject. The images display normal peripapillary RNFL thickness maps, quadrant-specific thickness values, and optic disc topography, with color-coded maps showing RNFL thickness within normal limits. No areas of abnormal thinning are observed, and the RNFL symmetry and quadrant measurements fall within the expected physiological range for a healthy subject.

RNFL thickness was analysed using spectral domain OCT (RS-3000 Advance (NIDEK Co., Ltd., Gamagori, Aichi, Japan)) following pupil dilation. Fundus surface images were captured with a confocal laser scanning ophthalmoscope using a near-infrared light at a wavelength of 785 nm, while cross-sectional retinal images were obtained via optical interferometer using an infrared light source at 880 nm. The used OCT scanning mode was disc circle mode (slice distance 3.45 mm; 1024 scans), in which the optic disc was scanned in a circular pattern following the order “Temporal”, “Superior”, “Nasal”, and “Inferior”. This allowed for the acquisition of OCT images to evaluate the retina. The average peripapillary RNFL thickness (measured from the inner limiting membrane (ILM) to the nerve fiber layer (NFL) or ganglion cell layer (GCL)) and quadrant-specific thickness were calculated. Additionally, the macular ganglion cell layer (GCL) thickness was measured in the central 1 mm zone, the inner ring (diameter 1–3 mm from the central fovea), and in the nasal, superior, bitemporal, and inferior quadrants. OCT also automatically determined optic disc parameters, including disc area and rim area, with calculated values provided by the device. Figure 1 shows the OCT reading of PA patients. Figure 2 shows OCT reading of healthy subjects.

The optic chiasm thickness values were obtained by measuring the vertical diameter on the right side, left side, and the middle part. The deformations or signal changes of the optic chiasm were evaluated and documented. The distance between the superior margin of the tumor and the inferior surface of the optic chiasm was measured as well.

### 2.4. Brain Imaging

**Figure 3:**
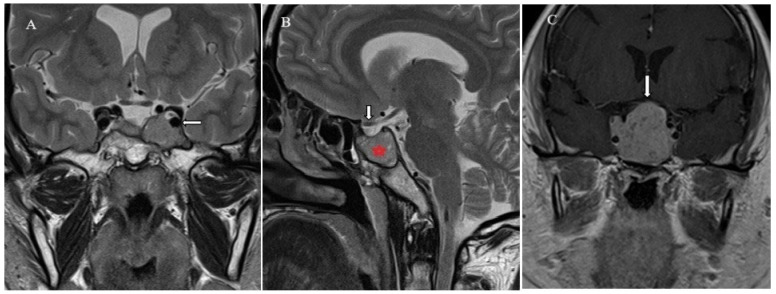
MRI images. (**A**) Coronal and (**B**) sagittal T2-weighted MR images show an isointense mass, which expands the left adenohypophysis (asterisk) and encases the carotid arteries (long arrow) but does not compress the optic chiasm (short arrow). (**C**) Coronal contrast-enhanced T1-weighted MR image shows mild homogeneous enhancement of the giant pituitary macroadenoma with elevation and compression of the optic chiasm (arrow indicates the area of optic chiasm).

All PAs were evaluated based on MRI findings (Figure 3). Preoperative MRI scans were performed using 1.5 T MRI scanners (Siemens MAGNETOM Avanto, 1.5 T (Siemens Healthcare, Erlangen, Germany)) with a head coil and a standard pituitary scanning protocol. This included T1-weighted sagittal and coronal images, T2-weighted/TSE coronal images before contrast, and T1-weighted coronal and sagittal images after contrast enhancement with gadodiamide (Omniscan, GE Healthcare, Chicago, IL, USA). A retrospective analysis of the MRI data was carried out by an experienced radiologist.

Pituitary adenomas were classified as macroadenomas if their maximum diameter was ≥10 mm and as microadenomas if <10 mm, based on preoperative MRI measurements.

### 2.5. Statistical Analysis

The statistical analysis was carried out using SPSS/W 30.0 software (Statistical Package for the Social Sciences for Windows, Inc., Chicago, IL, USA). The normality of data distribution was assessed using the Shapiro–Wilk and Kolmogorov–Smirnov tests. As most variables did not follow a normal distribution, non-parametric tests were applied. The Mann–Whitney U test was used to compare differences between independent groups. In this test, all values are ranked, and comparisons are based on the distribution of these ranks; the mean rank reflects the average position of values within each group, with a lower mean rank indicating generally lower measurements. Spearman correlation was used to assess the relationships between the thickness of the RNFL quadrants and the distance between the optic chiasm and the adenoma, and the height of the optic chiasm and VA. It was also used to examine the association between the thickness of the RNFL quadrants and optic chiasm height. A *p*-value of less than 0.05 was considered statistically significant.

### 2.6. Study Limitations

This study has several limitations that should be considered. Firstly, the small sample size (n = 25 for the PA group and n = 27 for the control group) may limit the statistical power and generalizability of the findings. A larger cohort would allow for more robust subgroup analyses and strengthen the reliability of observed associations. Secondly, because this study was cross-sectional, it only provides a snapshot at one point in time. This means we cannot determine cause-and-effect relationships or how structural changes seen on OCT relate to changes in visual function over time. Future studies that follow patients over a longer period are needed to understand how visual measures improve or worsen after treatment. Thirdly, although MRI and OCT assessments were conducted using standardized protocols, potential inter-observer variability in image acquisition and interpretation cannot be fully excluded and may have introduced measurement bias. Also, another limitation of this study is that giant pituitary adenomas were not investigated in detail, although they represent a distinct clinical group with more extensive chiasmal involvement. Future research should aim to evaluate the utility of OCT in this subgroup to better understand its role in predicting and monitoring visual outcomes. Additionally, the study did not control certain clinical variables, such as duration of symptoms, degree of visual impairment at presentation, or endocrine activity of the adenomas, which could have influenced the structural and functional metrics. Furthermore, this study involved multiple comparisons across RNFL and GCL quadrants and subgroups, which may increase the risk of Type I errors. While a significance threshold of *p* < 0.05 was used throughout, no formal correction for multiple testing (e.g., Bonferroni) was applied, given the exploratory nature of the study and the limited sample size. As such, findings that reached statistical significance in isolated quadrants or subgroups should be interpreted with caution. These preliminary observations provide direction for future research, which should include multicenter prospective studies with larger and more clinically homogeneous populations to validate and expand upon these findings.

## 3. Results

Our research included 25 patients (50 eyes) diagnosed with PA by MRI and 27 (54 eyes) subjects as healthy controls. The PA and control groups showed no significant differences in age (median (min–max): 47 (24–55) vs. 52 (26–70); *p* = 0.072) or gender distribution (males: 14 (28%) vs. 12 (22.2%), females: 36 (72%) vs. 42 (77.8%); *p* = 0.497).

### 3.1. Visual Acuity Changes

The median VA in PA patients was lower than in control group 1 ((min 0.01; max 1), mean rank 48.18 vs. 1 (min 1; max 1); mean rank 56.5; *p* = 0.002). VA in the group with suprasellar PA extension (mean rank: 23.14) and without it (mean rank: 28.5) differed statistically significantly (*p* = 0.043) (Table 1).

### 3.2. Visual Field Defects

A normal visual field was found in 28 eyes (56%) among the PA patients; no VF defects were found in control group eyes.

### 3.3. Optical Coherent Tomography

RNFL thickness around the optic disk measured preoperatively was reduced significantly in the temporal quadrant in PA patients compared with the control group (median (min; max); mean rank: 73.5 (52; 109); 58.39 vs. 69.5 (16; 168); 46.14; *p* = 0.038). Comparing the thicknesses of the GCL+ and GCL++ layers in the study groups, we found no statistically significant differences. Similarly, no statistically significant differences were found when assessing the thickness of these layers in the two sectors (superior and inferior) (*p* > 0.05) (Table 2).

GCL++ thickness around the optic disk was measured in the micro and macro-PA groups. We found it was reduced significantly only in the inferior sectors of the macro-PA group compared to the micro-PA group (median (min; max); mean rank: 99.5 (61; 115); 21.07 vs. 106.5 (90; 121); 32.15), *p* = 0.008, respectively) (Table 3).

RNFL, GCL+ and GCL++ thickness around the optic disk were measured in invasive and non-invasive PA groups. Unfortunately, there were no statistically significant results (all *p* > 0.05) (Table 4).

Continuing the research, the RNFL, GCL+ and GCL++ thickness values around the optic disk were measured in the active and non-active PA groups. We found that RNFL thickness was reduced significantly only in the inferior quadrant of the non-active PA group compared to the active PA group (median (min; max); mean rank: 118.5 (49; 144); 17.42 vs. 130.5 (77; 156); 28.05), *p* = 0.028, respectively) (Table 5).

RNFL, GCL+ and GCL++ thickness was measured in the PA with suprasellar extension group and the PA without suprasellar extension group. We found that RNFL thickness was reduced significantly only in the temporal quadrant in the PA with suprasellar extension group compared to the PA without suprasellar extension group (median (min; max); mean rank: 67.5 (16; 99); 21.66 vs. 72 (58; 168); 30.39), *p* = 0.036, respectively). Furthermore, GCL++ thickness was reduced significantly in both superior and inferior sectors in all of the PA with suprasellar extension group compared to the PA without suprasellar extension group (median (min; max); mean rank: 98.5 (57; 113); 21.8; 101 (61; 121); 21.48 and 102.5 (59; 116); 21.71 vs. 103.5 (95; 115); 30.2; 106.5 (90; 115); 30.61 and 104.5 (95; 113); 30.32), *p* = 0.043; *p* = 0.028 and *p* = 0.038, respectively) (Table 6).

In both the patient and control groups, the correlations between the GCL+ and GCL++ with disc and rim areas were assessed. However, at this stage of the analysis, no statistically significant associations were observed (Table 7).

### 3.4. Correlation Analysis Between Optic Disc and Rim Areas with RNFL Thickness

Table 8 presents the correlation between optic disc area and optic rim area with RNFL thickness across different quadrants in both patients with PA and the control groups.

### 3.5. Visual Field Correlations with RNFL

Table 9 presents the correlation between VF defects—measured as the number of undetected points in a 135-point full-field perimetry—and RNFL thickness across different quadrants in patients with PA.

The results reveal moderate to strong negative correlations between VF defects and RNFL thickness across all quadrants. The strongest correlation was found in the superior quadrant (r = −0.618, *p* < 0.001), followed by the total RNFL thickness (r = −0.552, *p* < 0.001). The temporal, inferior, and nasal quadrants also showed significant negative correlations, though weaker in magnitude (r = −0.316 to −0.434). All correlations reached statistical significance, suggesting a meaningful relationship between RNFL thinning and increased visual field loss.

These findings indicate that in patients with PA, a thinner RNFL is associated with greater visual field defects, highlighting the clinical relevance of structural OCT measurements in assessing visual function impairment.

### 3.6. Optic Disc Area Correlations

Our study analysis showed no significant correlations between optic disc area and RNFL thickness across all quadrants in the PA group (*p* > 0.05).

In the control group, significant positive correlations were found between optic disc area and total RNFL thickness (r = 0.440, *p* < 0.001), and a similar correlation between optic disc area and RNFL thickness was observed in the superior quadrant (r = 0.426, *p* = 0.001). However, the correlation was weaker for the temporal (*p* = 0.06) and nasal quadrants (*p* = 0.142), with only the inferior quadrant showing significance (r = 0.350, *p* = 0.009) (Table 8).

### 3.7. Optic Rim Area Correlations

In the PA group, significant correlations were observed between optic rim area and total RNFL thickness (r = 0.493, *p* < 0.001) in all quadrants, with the strongest being in the nasal quadrant (r = 0.503, *p* < 0.001).

In the control group, significant correlations were also observed between optic rim area and total RNFL thickness (r = 0.351, *p* = 0.009) across the superior (r = 0.333, *p* = 0.014), temporal (r = 0.447, *p* < 0.001), and inferior (r = 0.334, *p* = 0.014) quadrants, except for the nasal quadrant (r = −0.065, *p* = 0.641), indicating no correlation (Table 8).

## 4. Discussion

The thinning of the RNFL usually correlates directly with visual disturbances and the extent of chiasm compression [19]. The median VA in PA patients was lower than in the control group 1 (min 0.01; max 1), with a mean rank of 48.18 vs. 1 (min 1; max 1) and a mean rank of 56.5; *p* = 0.002. VA in the group with suprasellar PA extension (mean rank: 23.14) and without it (mean rank: 28.5) differed statistically significantly (*p* = 0.043), and VA decreased in 10% of cases in our research. In the literature, we can observe that patients presented with VA complaints in 14–84% of PA cases [20,21]. In our research, abnormal VF was found in 44% of PA patients. In research carried out by Lee and colleagues, a total of 89 of 115 patients had an abnormal VF. Only one patient had true bitemporal hemianopsia (BHA). The most common defects were bitemporal or mixed defects (in 49 of 115 patients 42.6%), likely because more than just the chiasm is often compressed by macro-PA [22]. Anderson and others reported that only 16% of their patients had decreased VA, and 32% had VF defects [23].

PAs and other suprasellar tumors can lead to thinning of the RNFL, due to their compressive effect on the optic chiasm. Preoperative RNFL thickness has been reported to be an important prognostic factor in predicting visual recovery after surgery [24,25,26,27]. Therefore, prolonged compression of the visual pathway leads to a progressive loss of ganglion cells and retinal nerve fibers. OCT enables a quantitative assessment of RNFL and GCL thinning and is therefore a valuable prognostic tool [28,29]. In the study by Iqbal et al., preoperatively, 19 (47.5%) eyes had RNFL thickness of <85 μm, 13 (32.5%) eyes had RNFL thickness of between 85 μm and 105 μm, and 8 (20%) had thickness of >105 μm [30]. In our study group, RNFL was between 40 μm and 129 μm, and total RNFL was 99 μm in PA patients. Our results are consistent with previous studies, demonstrating that preoperative RNFL thickness in the temporal quadrant around the optic nerve head was significantly lower in PA patients compared to the controls (median (min; max); mean rank: 73.5 (52; 109); 58.39 vs. 69.5 (16; 168); 46.14; *p* = 0.038). It is generally reported that the thickest RNFL measurements are found in the inferior quadrant, followed by the superior, nasal, and temporal quadrants [16,24,26,28,31,32]. However, for instance, Monteiro et al. did not observe greater RNFL thinning in the temporal and nasal quadrants compared to the vertical quadrants [33]. As the decussating nasal fibers are located in the middle, tumors pressing on the optic chiasm preferentially compress the decussating nasal fibers, resulting in retrograde RNFL thinning in both the nasal and temporal areas of the optic disc [34].

Importantly, our study revealed moderate to strong negative correlations between VF defects and RNFL thickness across all quadrants. The strongest correlation was found in the superior quadrant (r = −0.618, *p* < 0.001), followed by the total RNFL thickness (r = −0.552, *p* < 0.001). All correlations reached statistical significance, suggesting a meaningful relationship between RNFL thinning and increased VF loss. These findings indicate that in patients with PA, a thinner RNFL is associated with greater VF defects, highlighting the clinical relevance of structural OCT measurements in assessing visual function impairment.

Tieger and colleagues state that patterns of GCL thinning observed on OCT may help to localize compressive lesions in the anterior visual pathway, particularly in cases involving chiasm compression. Detecting GCL thinning on OCT can serve as an early indicator of visual pathway damage, potentially appearing before RNFL thinning or even VA loss. However, in our study, no statistically significant differences were found when comparing the thicknesses of the GCL+ and GCL++ layers between groups [7]. In contrast, Pang et al. reported that the nasal, superior, and inferior GCL regions were thinner in PA patients compared to controls [8], a finding consistent with Yum HR et al. [9], who observed reduced macular GCL and inner plexiform layer (GCIPL) thickness in the superior, inferior, and nasal regions of the macula in PA patients compared to controls. Poczos et al. found that the nasal and temporal GCL values of PA patients with severe optic nerve compression before surgery were thinner than those of the control group [10]. We also evaluated GCL++ thickness around the optic disk, which was measured in micro and macro-PA groups. We found it was reduced significantly only in the inferior sector of the macro-PA group compared to micro-PA (median (min; max); mean rank: 99.5 (61; 115); 21.07 vs. 106.5 (90; 121); 32.15, *p* = 0.008, respectively). Furthermore, we found that RNFL thickness was reduced significantly only in the inferior quadrant of non-active PA group compared to active PA (median (min; max); mean rank: 118.5 (49; 144); 17.42 vs. 130.5 (77; 156); 28.05, *p* = 0.028, respectively).

We found a significant reduction in RNFL thickness specifically in the temporal quadrant in PA patients with suprasellar extension compared to PA patients without suprasellar extension (median (min; max); mean rank: 67.5 (16; 99); 21.66 vs. 72 (58; 168); 30.39, *p* = 0.036, respectively). Furthermore, GCL++ thickness was reduced significantly in both the superior and inferior sectors and the total of the PA with suprasellar extension group compared to the PA without suprasellar extension group (median (min; max); mean rank: 98.5 (57; 113); 21.8; 101 (61; 121); 21.48 and 102.5 (59; 116); 21.71 vs. 103.5 (95; 115); 30.2; 106.5 (90; 115); 30.61 and 104.5 (95; 113); 30.32; *p* = 0.043; *p* = 0.028 and *p* = 0.038, respectively). In the study by Glebauskiene et al., the thickness of the optic chiasm was significantly different between patients with and without suprasellar PA extension (*p* < 0.001) [16]. Additionally, RNFL thickness was significantly reduced only in the temporal quadrant in PA patients with suprasellar extension compared to patients without suprasellar extension (*p* = 0.009) [16]. Lei et al. studied 44 patients with PA (21 without and 23 with chiasmal compression) and 18 healthy controls. Using SD-OCT, they measured RNFL and GCL thickness across all participants [35]. The three groups (PAs with chiasmal optic nerve compression, PAs without chiasmal optic nerve compression, and controls) were comparable in terms of mean age, gender, and intraocular pressure (*p* = 0.173, *p* = 0.184 and *p* = 0.343, respectively). However, both RNFL and GCL thickness differed significantly between the three groups (RNFL: 94.1 ± 12.5 µm, 106.4 ± 7.3 µm, 110.7 ± 6.9 µm, and GCL: 85.8 ± 6.9 µm, 93.8 ± 5.0 µm, 97.2 ± 5.6 µm, respectively). Regional RNFL analysis revealed that even in the absence of visible chiasmal compression, patients with PA showed significant thinning in the superior and inferior nasal regions compared to the control group [35].

The clinical value of OCT is its high sensitivity for detecting early structural changes in the visual pathway, particularly RNFL thinning, even when chiasmal compression is minimal or not yet radiologically evident. This early detection capability enables timely diagnosis and intervention, which is critical for preventing irreversible visual impairment [27]. Unlike VF testing, which detects functional deficits, OCT can reveal subclinical damage by identifying thinning of the RNFL before measurable vision loss occurs. This makes OCT a more sensitive and objective method for monitoring disease progression and guiding surgical decision making. As such, these findings emphasize the role of OCT as an essential component of preoperative evaluation in all patients with PAs, providing valuable insight into the integrity of the visual pathway and helping clinicians to assess the urgency and potential benefit of treatment [36].

This study highlights the potential of OCT-derived RNFL and GCL measurements as structural biomarkers for assessing visual pathway involvement in patients with PAs. The findings underscore the importance of temporal RNFL thinning in cases with suprasellar extension and suggest that the optic rim area may be a more reliable indicator of RNFL changes than the disc area. Future studies should aim to validate these results in larger cohorts and assess longitudinal changes pre- and post-operatively to determine the predictive value of OCT parameters for visual recovery. Further research may also explore the integration of OCT with advanced MRI techniques to improve diagnostic accuracy and enable earlier intervention. These insights could contribute to the development of more precise, individualized treatment strategies for patients with PA.

## 5. Conclusions

Our study discusses PA’s impact on RNFL, GCL thickness, and visual function due to compression of the optic chiasm. VA was significantly lower in PA patients, particularly in those with suprasellar extension, and many presented with VF defects. OCT was highlighted as a crucial tool for detecting RNFL thinning, which correlates with visual disturbances and can help predict visual recovery after surgery. The findings align with previous research showing that RNFL thinning occurs mainly in the temporal quadrant in PA patients with suprasellar extension. GCL thinning was observed but was not statistically significant in all cases. Our correlation analysis demonstrates a significant negative relationship between RNFL thickness and visual field defects, reinforcing the clinical relevance of OCT in visual function assessment. Furthermore, our findings suggest that optic rim area may be a more reliable indicator of RNFL thickness variations than optic disc area in the PA group. Our findings highlight the potential of OCT as a valuable prognostic tool for patients with PA, offering a non-invasive method to detect structural changes in the retina that correlate with visual function and disease severity. Incorporating OCT into routine clinical assessments could help to stratify patients based on their risk of visual impairment, enabling earlier intervention and tailored follow-up strategies. Furthermore, the ability to monitor retinal changes over time may support treatment decisions and assess response to therapy. This approach could enhance personalized care by identifying patients who may benefit from more aggressive management or closer surveillance. Ultimately, integrating OCT-derived biomarkers into clinical practice has the potential to improve outcomes by guiding prognosis and optimizing therapeutic strategies for patients with PAs.

## Figures and Tables

**Table 1:** Demographic and clinical data.

Characteristics		Group		*p*-Value
		PA Group, n = 25	Control Group, n = 27	
**Gender**	Males, N (%)	14 (28)	12 (22.2)	0.497
	Females, N (%)	36 (72)	42 (77.8)	
**Age min–max, median**		24–55, 47	26–70, 52	0.072
**Size**	Micro PA	10 (40)	-	-
	Macro PA	15 (60)		
**Invasiness**	Invasive PA	15 (60)		
	Non-invasive PA	10 (40)		
**Activeness**	Active Pa	19 (76)		
	Non-active PA	6 (24)		
**Symptoms Per Eye**				
**Visual disturbances (decreased visual acuity)**				
-Decreased		5 (10)	0 (0)	**0.017**
-Not decreased		45 (90)	54 (100)	
**Visual acuity of all PA patients** median (min; max); mean rank		1 (0.01; 1); 48.18	1 (1; 1); 56.5	**0.002**
-PA with suprasellar extension median (min; max); mean rank		1 (0.01; 1); 23.14	-	**0.043**
-PA without suprasellar extension median (min; max); mean rank		1 (0.8; 1); 28.5		
**Visual disturbances (visual field defects)**				
-Detected		22 (44)	0 (0)	<0.001
-Not detected		28 (56)	54 (100)	

PA—pituitary adenoma; Significant *p* values are bolded.

**Table 2:** Retinal nerve fibre layer (RNFL) and ganglion cell layer (GCL) changes per eye in patients with PA and control group subjects.

RNFL Quadrants	Control Group: Median (Min; Max); Mean Rank (n = 54)	PA Group: Median (Min; Max); Mean Rank (n = 50)	*p*-Value *
Temporal quadrant	73.5 (52; 109); 58.39	69.5 (16; 168); 46.14	**0.038**
Superior quadrant	126.5 (67; 165); 56.76	120 (57; 156); 47.9	0.134
Nasal quadrant	78 (50; 119); 56.34	75 (36; 119); 48.35	0.177
Inferior quadrant	130.5 (104; 1798); 56.28	129 (49; 156); 48.42	0.184
Total	102 (86; 125); 56.16	99 (40; 129); 48.55	0.198
**GCL+ (Ganglion Cell Layer [GCL] + Inner Plexiform Layer [IPL])**	**Control Group: Median (Min; Max); Mean Rank (n = 54)**	**PA Group: Median (Min; Max); Mean Rank (n = 50)**	*p*-Value *
Superior	65 (56; 74); 56.8	62.5 (44; 72); 47.86	0.130
Inferior	64.5 (55; 77); 55.66	62 (42; 76); 49.09	0.266
Total	65 (54; 74); 56.82	62 (43; 71); 47.83	0.128
**GCL++ (RNFL + GCL + IPL)**	**Control Group: Median (Min; Max); Mean Rank (n = 54)**	**PA Group: Median (Min; Max); Mean Rank (n = 50)**	*p*-Value *
Superior	104 (69; 116); 54.37	102 (57; 115); 50.48	0.510
Inferior	105 (93; 126); 57.54	105 (61; 121); 47.06	0.076
Total	104 (92; 117); 56.37	103.5 (59; 116); 48.32	0.173

* Mann–Whitney U test; Significant *p* values are bolded.

**Table 3:** Retinal nerve fiber layer (RNFL) and ganglion cell layer (GCL) changes in patients with microadenoma and macroadenoma.

RNFL Thickness, μm	Microadenoma: Median (Min; Max); Mean Rank (n = 20)	Macrodenoma: Median (Min; Max); Mean Rank (n = 30)	*p*-Value *
Temporal quadrant	71 (58; 142); 29.38	69 (16; 168); 22.92	0.125
Superior quadrant	120 (95; 156); 26.9	120 (57; 144); 24.57	0.579
Nasal quadrant	75.5 (46; 119); 29.65	69.5 (36; 107); 22.73	0.100
Inferior quadrant	130.5 (99; 152); 27.68	125.5 (49; 156); 24.05	0.389
Total	101.5 (81;129); 28.95	99 (40; 118); 23.2	0.171
**GCL+ (Ganglion Cell Layer [GCL] + Inner Plexiform Layer [IPL])**	**Microadenoma: Median (Min; Max); Mean Rank (n = 20)**	**Macrodenoma: Median (Min; Max); Mean Rank (n = 30)**	*p*-Value *
Superior	62.5 (53; 68); 26.38	62.5 (44; 72); 24.92	0.728
Inferior	63 (56; 76); 28.35	62 (42; 69); 23.60	0.258
Total	62.5 (54; 69); 26.65	62 (43; 71); 24.73	0.648
**GCL++ (RNFL + GCL + IPL)me**	**Microadenoma: Median (Min; Max); Mean Rank (n = 20)**	**Macrodenoma: Median (Min; Max); Mean Rank (n = 30)**	*p*-Value *
Superior	102.5 (98; 112); 28.08	99.5 (57; 115); 23.78	0.307
Inferior	106.5 (90; 121); 32.15	99.5 (61; 115); 21.07	**0.008**
Total	104 (95; 116); 29.65	100 (59; 113); 22.73	0.100

* Mann–Whitney U test; Significant *p* values are bolded.

**Table 4:** Retinal nerve fiber layer (RNFL) and ganglion cell layer (GCL) changes in patients per eye in cases of invasive and non-invasive pituitary adenoma.

RNFL Thickness, μm	Invasive PA: Median (Min; Max); Mean Rank (n = 30)	Non-Invasive PA: Median (Min; Max); Mean Rank (n = 20)	*p*-Value *
Temporal quadrant	72.5 (16; 168); 26.5	68 (28; 159); 24	0.552
Superior quadrant	120 (57; 156); 25.3	120 (67; 144); 25.8	0.905
Nasal quadrant	74 (36; 107); 23.83	75.5 (46; 119); 28	0.322
Inferior quadrant	126.5 (49; 156); 23	132.5 (77; 154); 29.25	0.137
Total	99 (40; 118); 24.82	101.5 (63; 129); 26.53	0.684
**GCL+ (Ganglion Cell Layer [GCL] + Inner Plexiform Layer [IPL])**	**Invasive PA: Median (Min; Max); Mean Rank (n= 30)**	**Non-Invasive PA: Median (Min; Max); Mean Rank (n = 20)**	*p*-Value *
Superior	63.5 (44; 72); 26.07	62 (50; 68); 24.65	0.735
Inferior	62.5 (42; 70); 24.9	62 (54; 76); 26.4	0.721
Total	63.5 (43; 71); 26.23	62 (52; 68); 24.4	0.662
**GCL++ (RNFL + GCL + IPL)**	**Invasive PA: Median (Min; Max); Mean Rank (n= 30)**	**Non-Invasive PA: Median (Min; Max); Mean Rank (n = 20)**	*p*-Value *
Superior	103.5 (57; 114); 26.17	101 (77; 115); 24.5	0.692
Inferior	104.5 (61; 121); 24.83	105.5 (78; 115); 26.5	0.692
Total	104 (59; 116); 26	102.5 (77; 113); 24.75	0.766

* Mann–Whitney U test.

**Table 5:** Retinal nerve fiber layer (RNFL) and ganglion cell layer (GCL) changes in patients (per eye) in cases of active and non-active pituitary adenoma.

RNFL Thickness, μm	Active PA: Median (Min; Max); Mean Rank (n = 38)	Non-Active PA: Median (Min; Max); Mean rank (n = 12)	*p*-Value *
Temporal quadrant	69 (28; 168); 25.86	71.5 (16; 86); 24.38	0.759
Superior quadrant	121 (67; 144); 27	113 (57; 156); 20.75	0.195
Nasal quadrant	77 (37; 119); 27.51	69.5 (36; 87); 19.13	0.082
Inferior quadrant	130.5 (77; 156); 28.05	118.5 (49; 144); 17.42	**0.028**
Total	104 (63; 129); 27.33	91 (40; 118); 19.71	0.114
**GCL+ (Ganglion Cell Layer [GCL] + Inner Plexiform Layer [IPL])**	**Active PA: Median (Min; Max); Mean Rank (n = 38)**	**Non-Active PA: Median (Min; Max); Mean Rank (n = 12)**	*p*-Value *
Superior	63.5 (46; 72); 27.2	61 (44; 70); 20.13	0.141
Inferior	62.5 (45; 70); 27.17	58.5 (42; 76); 20.21	0.148
Total	63 (46; 71); 27.64	56 (43; 68); 18.71	0.063
**GCL++ (RNFL + GCL + IPL)**	**Active PA: Median (Min; max); Mean Rank (n = 38)**	**Non-Active PA: Median (Min; Max); Mean Rank (n = 12)**	*p*-Value *
Superior	103 (71; 115); 27.16	100.5 (57; 111); 20.25	0.152
Inferior	105 (74; 121); 27.61	96.5 (61; 110); 18.83	0.069
Total	104 (73; 116); 27.33	98.5 (59; 110); 19.71	0.114

* Mann–Whitney U test; Significant *p* values are bolded.

**Table 6:** Retinal nerve fiber layer (RNFL) and ganglion cell layer (GCL) thickness in pituitary adenoma (PA) patients (per eye) with and without suprasellar extension.

RNFL Thickness, μm	PA with Suprasellar Extension: Mean Rank; Median (Min; Max) (n = 28)	PA Without Suprasellar Extension: Mean Rank; Median (Min; Max) (n = 22)	*p*-Value *
Superior quadrant	122.5 (57; 156); 25.55	120 (95; 144); 25.43	0.977
Temporal quadrant	67.5 (16; 99); 21.66	72 (58; 168); 30.39	**0.036**
Inferior quadrant	125 (49; 156); 22.23	131 (99; 154); 29.66	0.074
Nasal quadrant	71.5 (36; 95); 22.64	75.5 (46; 119); 29.14	0.118
Total	99 (40; 118); 23.2	100.5 (81; 129); 28.43	0.207
**GCL+ (Ganglion Cell Layer [GCL] + Inner Plexiform Layer [IPL])**	**PA with Suprasellar Extension: Mean Rank; Median (Min; Max)** **(n = 28)**	**PA Without Suprasellar Extension: Mean Rank; Median (Min; Max)** **(n = 22)**	*p*-Value *
Superior	63 (44; 70); 23.66	62 (53; 72); 27.84	0.312
Inferior	62 (42; 70); 22.46	63 (56; 76); 29.36	0.096
Total	62 (43; 69); 23.3	62.5 (54; 71); 28.3	0.228
**GCL++ (RNFL + GCL + IPL)**	**PA with Suprasellar Extension: Mean Rank; Median (Min; Max)** **(n = 28)**	**PA Without Suprasellar Extension: Mean Rank; Median (Min; Max)** **(n = 22)**	*p*-Value *
Superior	98.5 (57; 113); 21.8	103.5 (95; 115); 30.2	**0.043**
Inferior	101 (61; 121); 21.48	106.5 (90; 115); 30.61	**0.028**
Total	102.5 (59; 116); 21.71	104.5 (95; 113); 30.32	**0.038**

* Mann–Whitney U test; Significant *p* values are bolded.

**Table 7:** Correlation between optic disc area and ganglion cell layer (GCL+ and GCL++).

Versus	GCL+ (Ganglion Cell Layer [GCL] + Inner Plexiform Layer [IPL])			GCL++ (RNFL + GCL + IPL)		
	Total	Superior	Inferior	Total	Superior	Inferior
**Correlation Coefficient of Optic Disc Area Value in PA Group**	0.008	0.015	−0.025	−0.065	−0.129	−0.057
***p*-Value**	0.955	0.915	0.863	0.656	0.372	0.696
**Correlation Coefficient of Optic Disc Area Value in Control Group**	0.01	−0.14	0.064	−0.058	−0.114	−0.053
***p*-Value**	0.940	0.921	0.644	0.675	0.410	0.702
**Correlation Coefficient of Optic Rim Area Value in PA Group**	0.197	0.212	0.167	0.201	0.201	0.193
***p*-Value**	0.171	0.140	0.245	0.161	0.161	0.178
**Correlation Coefficient of Optic Rim Area Value in Control Group**	0.136	0.106	0.122	−0.109	−0.113	−0.146
***p*-Value**	0.328	0.447	0.379	0.434	0.417	0.293

**Table 8:** Correlation between optic disc area/rim area and retinal nerve fiber layer (RNFL) thickness.

Versus	RNFL Thickness				
	Total	Superior Quadrant	Temporal Quadrant	Inferior Quadrant	Nasal Quadrant
**Correlation Coefficient of Optic Disc Area Value in PA Group**	0.046	0.094	−0.011	−0.012	0.153
***p*-Value**	0.751	0.716	0.937	0.935	0.288
**Correlation Coefficient of Optic Disc Area Value in Control Group**	0.440	0.426	0.268	0.350	0.203
***p*-Value**	**<0.001**	**0.001**	**0.06**	**0.009**	0.142
**Correlation Coefficient of Optic Rim Area Value in PA Group**	0.493	0.295	0.327	0.368	0.503
***p*-Value**	**<0.001**	**0.037**	**0.020**	**0.008**	**<0.001**
**Correlation Coefficient of Optic Rim Area Value in Control Group**	0.351	0.333	0.447	0.334	−0.065
***p*-Value**	**0.009**	**0.014**	**<0.001**	**0.014**	0.641

Significant *p* values are bolded.

**Table 9:** Correlation between visual field (VF) and retinal nerve fiber layer (RNFL) thickness.

Versus	RNFL Thickness				
	Total	Superior Quadrant	Temporal Quadrant	Inferior Quadrant	Nasal Quadrant
**Correlation Coefficient of Visual Field Value in PA Group**	−0.552	−0.618	−0.316	−0.434	−0.282
***p*-Value**	**<0.001**	**<0.001**	**0.025**	**0.002**	**0.047**

Significant *p* values are bolded.

## Data Availability

The datasets used and/or analyzed during the current study are available from the corresponding author on reasonable request.
